# Spatial omics and its applications for a tumour microenvironment characterization and precision medicine approach of gastric cancer

**DOI:** 10.3332/ecancer.2026.2140

**Published:** 2026-06-04

**Authors:** José Darío Portillo-Miño, John Jairo Calderon, David Bettin Gonzalez, Yeison Carlosama, Lorena Lagarde, Rafael Parra-Medina

**Affiliations:** 1 Division of Research, Instituto Departamental de Salud de Nariño (IDSN), Pasto 520003, Colombia; 2 Epidemiology Master Program, Department of Public Health and Epidemiology, Faculty of Health Sciences, Pontificia Universidad Javeriana, Cali 760031, Colombia; 3 Grupo de Investigación en Ciencia y Salud (GICIENSA), Faculty of Health Sciences, Medicine Program, Universidad de Nariño, Pasto 520001 Colombia; 4 Hospital Universitario Departamental de Nariño, Pasto 520003, Colombia; 5 Grupo de Investigación Ciencia y Salud (GICIENSA), School of Medicine, Universidad de Nariño, Pasto, Nariño, Colombia; 6 Biological Sciences Master Program, Faculty of Sciences, Pontificia Universidad Javeriana, Bogota 110231, Colombia; 7 Grupo Interdisciplinario de Investigación en Salud y Enfermedad (GIISE), Universidad Cooperativa de Colombia, Pasto, Colombia; 8 Digestive Oncology High-Specialist Resident Program, Instituto Nacional de Cancerología, México City 14080, México; 9 Grupo de Patología y Oncología Molecular, Instituto Nacional de Cancerología (INC), Bogotá 111511, Colombia; 10 Research Institute, Fundación Universitaria de Ciencias de la Salud - FUCS, Bogotá 111411, Colombia

**Keywords:** gastric cancer, tumour microenvironment, spatial omics, proteomics, genomic, transcriptomic

## Abstract

Gastric cancer (GC) is a lethal disease due to heterogeneity and complexity from the point of view of epidemiology, epidemiology and public health policy and biological behaviour. Despite recent advances in the research field on the molecular and cellular characterisation of GC, the clinical outcomes have been ominous. Perhaps the unceasing research in future years, focusing on spatial omics, has revolutionary potential for transforming the conception and understanding of GC mechanisms, thereby enabling the discovery of targeted therapies that are most effective in improving survival. Under these circumstances, this review article lays the groundwork for an update of spatial omics, offering a comprehensive vision and its implications for understanding the landscape of GC research. Moreover, it highlights the recent advances and breakthroughs in heterogeneity and immunosuppressive tumour microenvironment.

## Introduction

Gastric (stomach) cancer (GC) is well-known for leading to the mortality of cancer globally since it is considered a fatal disease, with a mortality projected of 832.310 by 2050 [1]. However, research efforts have emerged in this field. Nevertheless, aspects of cell and molecular biology, epidemiologic patterns dimensions of clinical complexity and heterogeneity of the disease contribute to the worst survival rates [2–6]. The urgency to develop novel and effective therapies to counteract this disease is paramount. In this sense, technological advances in the omics sciences enable breakthroughs through platforms such as proteomics and next-generation sequencing (NGS), with the capacity to characterise molecular and cellular subtypes of GC, thereby enhancing our understanding of GC biology and cellular immunobiology [2].

Furthermore, this allows it to complement the histopathological classifications of the Lauren system (diffuse and intestinal), Nakamura (differentiated and undifferentiated) and the World Health Organisation, such as mucinous, papillary, poorly cohesive and tubular [3–5]. A recent approach to molecular characterisation, The Cancer Genome Atlas (TCGA), has been a significant milestone in understanding the molecular and cellular biology of GC and characterising it into four subtypes: Epstein-Barr virus (EBV), microsatellite instability (MSI), genomically low stable (GS) and chromosome instability (CIN) [4]. The impact of this project is evident in the development of novel targeted therapies, such as PDL-1, CTLA4 and HER2 response, that have improved partial clinical outcomes, bringing us closer to a more appropriate and precise classification of GC prediction and behaviour.

The diagnosis is made by esophagogastroduodenoscopy with biopsy, according to the Sydney classification, which allows for histopathological examination [6–10]. Moreover, the staging of the disease involved computed tomography, magnetic resonance imaging, ultrasound, laparoscopy and fluoro-deoxy-glucose-proton emission tomography [9, 10].

In recent years, omics science has paved the way for developing novel therapies to understand the tumour microenvironment (TME); with its multiple cell-cell interactions and dynamic communications between diverse cell populations and signaling pathways, this ecosystem is a crucial focus for redirecting the understanding of GC [11, 12]. Given this scenario, providing current omics-based classifications can facilitate enhanced prediction of responses to immunotherapy and novel therapies in development for GC [13]; even translating basic tumour biology into clinical application can pose a challenge to effective cancer treatments [13–16]. Through NGS technologies, proteomics and genomics, essential details about GC’s origins have been uncovered, offering new insights. In this sense, recent studies have revealed complex dynamics surrounding cell-cell interactions and the cellular communication network within their TME [2, 11, 12]. Additionally, the interconnections between transcriptomics and genomics are significant, as they reveal genomic and transcriptomic cluster subtypes that encompass epithelial-mesenchymal transition (EMT), metabolic and immune hallmarks [2, 10].

Given this challenge, the authors in this review article provide an update on spatial omics, offering a comprehensive view of the implications for understanding the GC research landscape. Moreover, it highlights the recent advances and breakthroughs in the immunosuppressive and diversity of TME in GC.

## GC molecular classification - the journey begins

TCGA has been a significant milestone in understanding the molecular and cellular biology of GC. This project has allowed us to characterise gastric adenocarcinoma into four subtypes: EBV, MSI, GS and CIN [2]. The impact of this project is evident in the development of novel targeted therapies that have improved partial clinical outcomes. Another vital component in understanding GC is Lei’s classification, which divides GC into metabolic, proliferative and mesenchymal (MES) subtypes. This classification allows us to assess chemotherapy response based on integrated biological properties [21]. In addition, the gene expression profile carried out by the Asian Cancer Research Group showed four subtypes of GC: MSI, MSI/EMT, microsatellite stable/epithelial-TP53 loss (MSS/TP53-) and MSS/TP53+ without alteration [22].

The MSI is mainly present in the gastric antrum (75%) and intestinal subtype (60%), diagnosis is in early stages (>50%), showing high loss of MLH1 and high expression of DNA methylation, hypermutation of genes such as KRAS, ARID1A, ALK and PIK3-PTEN-mTOR signaling pathway [22, 23]. This MSI subtype is crucial in understanding GC because it is immune-sensitive and correlated with tumour-infiltrating lymphocytes (TILs) and PD-L1 expression [17].

The EBV subtype has been characterised by the overexpression of PIK3CA, ARID1A, CDKN2A, PDL-1/PDL2 and EBV-CIMP and is associated with immune sensitivity [2, 17].

**Figure 1.**
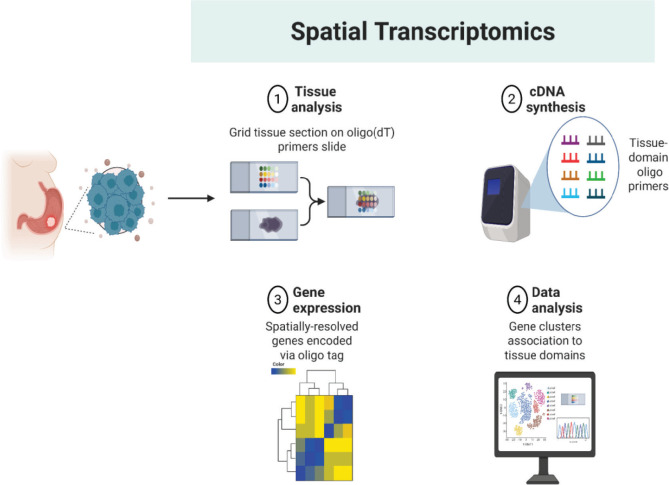
The general phases of spatialomics are described, in which the sample is extracted from the stomach to perform tissue analysis, then the synthesis of cDNA, the analysis of data on the association of the gene cluster with the tissue and finally, the genes expressed at the spatial level via oligo-tag.

The GS subtype is define by low somatic copy number alterations (SCNA) in the diffuse histological type and harbours mutations in RHOA1 (Ras homology family A1) and E-cadherin [2, 17]. Meanwhile, the CIN subtype exhibited mutation alterations in high PT53 with amplification of receptor tyrosine kinase-Ras [2, 17] (Figure 1). In summary, the molecular characterisation of GC has provided a deep understanding of the disease’s heterogeneity and complexity, despite its poor clinical outcomes.

Through NGS technologies, proteomics and genomics have revealed essential details in GC’s conception and novel insights. In this sense, recent studies have shown complex dynamics surrounding cell-cell interactions and the cellular communications network within their TME [17–20]. Additionally, the interconnections between transcriptomics and genomics are relevant, as they provide genomic and transcriptomic cluster subtypes that involve EMT, metabolic and immune hallmarks [12, 17]. This initial approach is significant because it lays the groundwork for emerging spatial omics research in GC.

## Approach of spatial-omics tools

### Spatial transcriptomic

The study of gene expression at the spatial level is exciting and challenging. Some aspects can address this. First, single-molecule fluorescence *in situ* hybridisation (smFISH) hybridisation revealed the spatial localisation of genes at single-cell resolution [11, 12]. The beneficial results of this method are that it performs a deep and comprehensive analysis of the spatial interplay and gene expression in unique cells within an exclusive tissue cross-section [22], with a low level of transcript detection, thereby enhancing sensitivity and subcellular resolution in spatial mapping [24, 25]. The limitations of the smFISH include that the overlap limits its ability to target multiple genes simultaneously in standard microscopes [11]. sequential fluorescence *in situ* hybridisation (SeqFISH) can visualise gene expression while maintaining an intact tissue structure, which is essential for spatial cell interactions [11, 26]. SeqFISH is an innovative technique that enables comprehensive gene detection, improving spatial resolution and sensitivity [11, 26].

Second, spatial transcriptomics technologies are based on high-throughput or NGS, which provides complete transcriptome data from whole tissue sections and additional spatial localisation information. Under these considerations, visual and cellular analyses provide data on the composition and elucidate the interplay of gene expression, aiming to identify new tissue biomarkers. Some technologies based on visual-spatial transcriptome sequencing include lymphocytic choriomeningitis (LCM)-RNA seq, high-definition spatial transcriptomics and slide-seq [11, 26–28].

Thirdly, the spatial transcriptomics technologies included fluorescence *in situ* sequencing, which allowed genotype discrimination within tissue cells and deciphered the TME on gene expression [30].

Fourth, this study focused on live-cell labeling; for example, transcriptome *in vivo* analysis plus RNA sequencing (RNA-seq) generated the most relevant information and comprehensive single-cell gene expression data. This method is exciting because it allows for the analysis of complex TME and tissue as a whole, utilising the single-cell transcriptome, but only for live-cell labeling, not frozen or fixed cells [25, 31]. On the other hand, Zip-seq is defined for using illumination patterns and photocaged oligonucleotides to attach DNA codes to live cells in unscathed tissues [32]. The szip codes can generate information on the spatial and contextual aspects of single-cell transcriptomic data [11, 33].

Integrating scRNA-seq and spatial transcriptomics involves characterising cell subpopulations within tissues and the spatial distribution networks of intercellular interactions and communications in their localisations [12]. This generates transcriptomic maps of tissues [34] and provides a comprehensive understanding of cell type distribution and potential mechanisms of cell-cell communication underlying tissue architecture [25]. Consistent with this idea, it can favour new inceptions into cell heterogeneity, immune response and functional and remodeling TME, which are critical for tumoural evolution [25, 35–38].

### Spatial proteomics

Spatial proteomics technology has demonstrated the ability to provide insight into analysing protein distribution in a wide array of tumour samples, thereby facilitating the detection of novel biomarkers and therapeutic targets [28, 39, 40]. In this scenario, this technology has enabled the collection of high-resolution data about the proteome in tumour tissues [28]. The exciting part is the comprehensive distribution and cell interaction of proteins in the TME [41].

Multiplex immunohistochemistry/immunofluorescence is a critical tool for detecting multiple and simultaneous proteins in the TME [11, 42]. The advantage of this approach is its capacity to understand spatial structures and the complex cell-cell and cell-protein interactions in the tumoural ecosystem [11, 42]. The imaging mass cytometry (IMC) and multiplexed ion beam imaging by the time of flight (MIBI-TOF) are technological tools that enhance the throughput of mass spectrometry [42–44]. The beneficial immunohistochemistry and mass spectrometry hybrid uses laser ablation for projected high-resolution images [42]. On the other hand, IMC preserves antigen specificity and reveals spatial distribution analysis [42–44].

The MIBI-TOF has been characterised for its use of ion mass spectrometry and isotopically labeled antibodies to project high-resolution images [42–44]. In this context, a notable example is Co-detection by indexing, which meets the conditions for utilising unique oligonucleotide sequence barcodes instead of enzyme reporter genes for multiplex cell counting imaging [42–45]. Matrix-assisted laser desorption/Ionisation-mass spectrometry imaging (MALDI-MSI) is an exciting option because it combines mass spectrometry with tissue microscopy, providing insight for *in situ* diagnosis and description of the cancer proteome and tumour clonal evolution over time [39, 46–49]. A relevant aspect of MALDI-MSI is the detection and localisation of proteins, causing holistic mapping [11, 25, 41, 42]. Moreover, it demonstrated high-resolution spatial mass spectrometric characteristics of interest cell types, thereby characterising heterogeneity in the TME. Protein microarray technology has enhanced the quantification and quality of spatial visualisation for tumour complexity and heterogeneity [17, 40, 41, 49, 50].

Lastly, other technologies are cutting-edge in the proteomic field, such as tissue-based cyclic immunofluorescence (t-CycIF), Immuno-SABER, MACSima imaging cyclic staging and deep visual proteomics [51, 52]. These technologies enable the mapping of detailed protein distribution and cellular subpopulations in the TME through protein expression patterns, pathological classifications and clinicopathological features [12, 53–55].

### Spatial metabolomics

Metabolomics is a fundamental tool that attempts to describe the spatial distribution of metabolites in the TME [56, 57]. This objective is feasible for MSI, which utilises label-free and matrix-free approaches with high sensitivity, enabling high-definition profiling of spatial metabolites and lipids surrounding the TME [58, 59]. Tracking the metabolites generates a mapping of the network and interplay between cells and metabolites [60, 61]. MALDI-MSI and desorption electrospray ionisation-mass spectrometry imaging (DESI-MSI) are two powerful tools that can identify compounds with significant molecular weight and hydrophobic, as well as lower isoelectric points, such as proteins and lipids. On the other hand, DESI-MSI utilises electrospray ionisation to extract metabolites through a fine mist of charged solvent droplets, bypassing the need for preparatory steps such as matrix application and enabling instantaneous analysis on the sample surface [56]. These characteristics make DESI-MSI accurate for *in situ* analysis, involving a speed advantage, comprehensive detection and low-mass metabolites analysis (lactate, glucose and amino acids) [11, 56]. This technology allows the identification and imaging of tumour-associated molecular alterations and cellular interactions of the tumour and its TME. The MSI explores tumoural spatial complexity to identify spatial distribution and metabolic patterns and detect novel targeted therapies [54, 60, 62].

### Artificial intelligence (AI) and its applications in spatial-omics

AI is a vigorous tool that helps understand the TME. It enables the analysis of data sources in combination with significant speed and capacity, generating insights into cellular heterogeneity and molecular interplay [63–65]. AI is characterised by deep learning, neuronal networks and the efficient processing of millions of complex data points, allowing for the classification of molecular subgroups, cellular states, intratumoural and intertumoural heterogeneity, tumour progression signal pathways and tumoural evolution [33, 63].

## Spatial-omics and new insights in gastric TME

The spatial-omics technology provides spatial information within tumour tissues, highlighting its applications in TME, tumour heterogeneity, tumour genesis and development mechanisms [66–68]. In this context, the potential contribution to medicine precision and disease understanding will facilitate an effective search for targeted therapies [13].

Spatial omics combines techniques from physics, informatics and computational science, aiming to exhibed the spatial distribution and interactions of cells, genes, RNA, proteins, metabolites, cytokines and chemokines with the sources of biological samples [24]. Additionally, this approach regains particular relevance due to its spatial distribution and localisation in three dimensions, enabling the simultaneous characterisation of the spatial structure and composition of tissues and cell populations within the TME, as well as multiplex analysis. This has demonstrated a high-throughput capacity and facilitated in-depth research [11]. The spatial omics technologies for GC included smFISH, LCM-seq, NanoString GeoMX, RNAScope, 10× Genomics Visium, MALDI-MSI, t-CycIF, IMC and DESI-MSI.

Spatial omics emerged due to tumour complexity and heterogeneity of GC, high cellular traffic and its multiple interconnections and communications with the canonical signaling pathways, and the competence of metabolites for survival in immunosuppressive and heterogeneous TME [14, 17, 18, 24].

Among the limitations of single-cell RNA-seq is the need to isolate tumour populations from their surrounding environment, which inherently produces cellular stress, alters cell mobilisation, replication or death processes and disrupts interactions with immune cells or fibroblasts. In short, this artificially modifies cellular physiology and plausibly genetic transcription. The primary advantages of spatial-resolved transcriptomics methods include the ability to assess gene transcription *in situ*, the reproducibility of spatial conditions and the study of cell-cell communication within cellular physiology. Some authors have studied the immunomodulation of the metastatic colorectal tumour phenotype through spatial transcriptomics [69], as well as the protumoural and peritumoural mechanisms of fibroblasts in the colorectal TME [70]; however, they have also been evaluated in gastric TME.

Spatial transcriptomics studies have been applied to assess tumour heterogeneity, characterise the TME and immune response, investigate mechanisms of neoplastic transformation, identify biomarkers and evaluate treatment response (Table 1, which provides a summary of the most relevant findings from key studies in this field). Moreover, it provides a systemic analysis of the tumour and its ecosystem within the TME [11, 24]. This tool focuses on analysing gene expression in the spatial context of cells and tissues, and its potential was highlighted in Nature Methods as the technique of the year in 2020 [71]. At this point, it is essential to remember that cell distribution and mapping in the TME will enable optimised immunotherapy and overcome pitfalls to improve clinical outcomes.

In the study conducted by Ma *et al* [72], an integrated model of 2,138 spatial transcriptomics regions of interest with 152,423 single-cell expression-based intratumoural heter described tumour progression through spatially localised and functionally ordered subgroups associated with tumour immune microenvironment (TIME) profiles, immune checkpoint profiles and genetic drivers (SOX9). In this research, the authors’ underscore spatial analysis of tumour–stroma interfaces across multiple GCs highlighted new ecosystem states not attributable to mere tumour/stroma admixture, landmarked by increased GREM1 expression. Moreover, immunosuppressive gene expression patterns in non-metastatic GC are crucial in the enriched-regulatory T cells (Treg) in gastric tumours; which contribute to immunosuppressive TME [73]. This study also observed the exhausted CD8+ T cells clusters, including inhibitory molecules such as PDCD1, CTLA4, HAVCR2, LAG-3 and TIGIT expression. MALD enables the detection of imaging tumour-associated molecular alterations and cellular interactions of tumours and cell types with its ecosystem TME at the metabolic level, intratumoural biochemical pathways and reprogramming in cancer growth and immune cells for the competence of surrounding substrates as a source of energy [11]. The metabolic barriers represent a challenge to cancer immunotherapy [74]; thus, if it can overcome the comprehensive characterisation of metabolite-cell interaction in the TME, this tool is fundamental for fulfilling that purpose.

**Figure 2.**
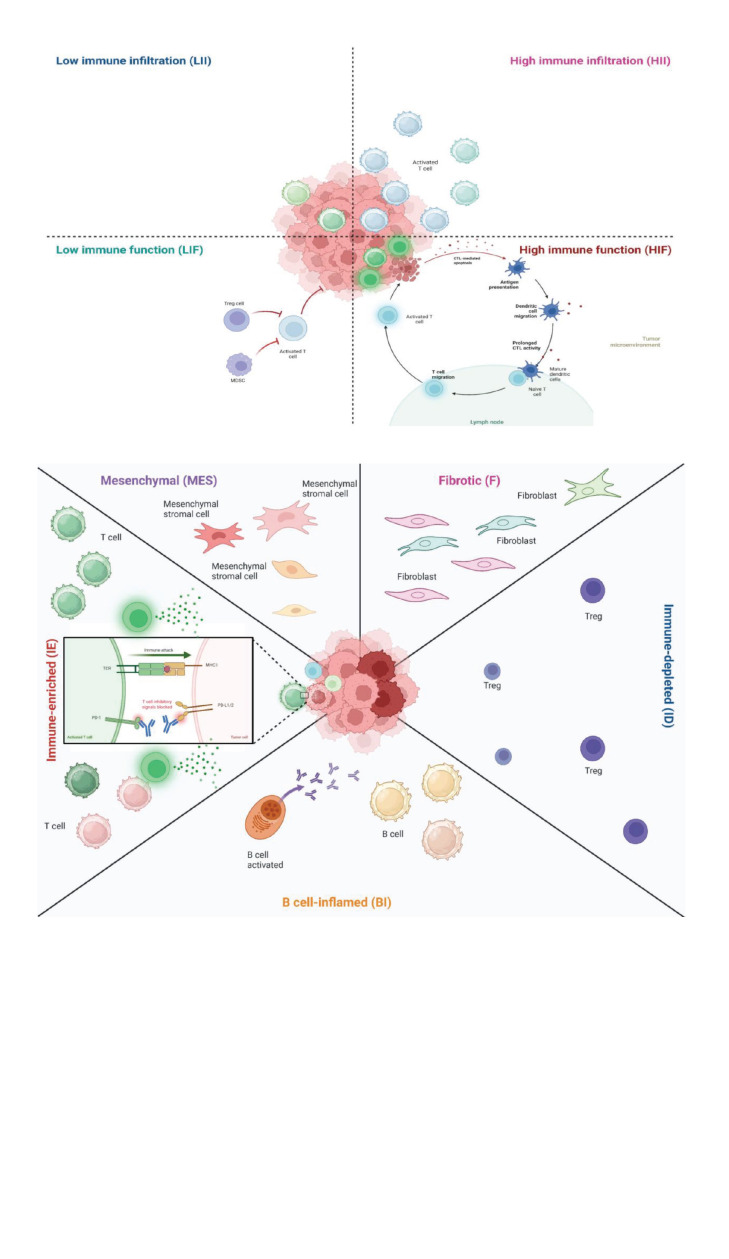
Approach of the immune characterisation using spatial omics platforms to immunotherapy response. (a): Cold and hot tumours have an immune response. Cold tumours are characterised by cytokines and chemokines that suppress immune responses, such as IL-10 and IL-5, which interact with immune cells [19]. (b):This characterisation proposes HIF, low immune function, HII and LII. Added to cold and hot tumours are complements to understanding with further precision, immune response and TME behaviour. In this context, GC MSI tumours are immune and sensitive to immunotherapy, responding to HIF and HII immune subtypes [53]. (c): This study was divided into five types: MES, F, IE, BI and immune-depleted (ID).This critical characterisation of gastric TME is crucial for understanding the immune response because it helps decipher the behaviour of GC and the immune response [81]. Created with www.bioRender.com.

On the other hand, it has been described as an interferon regulatory factor-8 downregulation in CD8+ TILs, which favours immunosuppressive TME [75]. This study, conducted using scRNA-seq, describes how critical inception in understanding TME reveals the immune landscape, cell subpopulations and T cells [76]. Another scRNA-seq study showed that high interferon regulatory factor-γ expression in CD8+ T cells was associated with enhanced responses to this combination therapy, suggesting the implications of the immune response on TME [77]. A study has examined the diversity of the TME landscape, which involves increased stromal cells and Treg cells, as well as exhausted CTL subtypes and a specific extracellular matrix (ECM) in TME [78].A study conducted by Yamasaki *et al* [79] using 10× Genomics Visium in organoids showed the critical role of hypoxia and mitogen-activated protein-kinase (MAPK) signaling in the metastatic progression of KRASG12V-expressing GC-p53KO tumours independent of Wingless/Integrated (WNT) signaling. For its part, Zheng *et al* [80] employed a novel scRNA-seq method, referred to as Visium 10× Genomics, which enables the description of cellular communication and interaction, as well as tumour heterogeneity. However, the limitation is that spatial data and interactions in these cellular subtypes are not provided. In this respect, the Derks *et al* [19] study reveals the TIME and immune subclasses distinct from gastric and esophagogastric adenocarcinoma (GEA) through the use of RNA-seq on a subset of cancers characterised in TCGA. This study is remarkable from the point of view of the immune system; it classified chromosomally unstable tumours into ‘cold’ and ‘hot’ categories, as **hot tumours** have a TME with high TILs, whereas **cold tumours** lack a favourable TME with TILs. This affected the response to immune checkpoint inhibitors (ICIs) and prognosis. In addition, the cold CIN-GEAs exhibited enrichment of myelocytomatosis oncogene and Cyclin E, whereas the E1 gene exhibited the hot CIN-GEAs [18, 19] (Figure 2A).

In this context, the study conducted by Shah *et al* [81] proposes TME-based molecular subtypes of GC that aim to predict immunotherapy response, clinical survival and prognosis. This study described five TME-based molecular subtypes, such as MES, fibrotic (F), immune-enriched (IE), B-cell inflamed (BI) and immune-depleted. Of these, MES and F are stromal-enriched courses with poor survival and prognosis, whereas IE with improved immune infiltration *T* cells are associated with the best prognosis (Figure 2B) [81].

The study conducted by Cabeza-Segura *et al* [53] in advanced GEA revealed immune cluster subtypes and signaling related to immunomodulatory pathways; in this respect, these subtypes exposed enriched immune infiltrate in two groups with high (HII) and low (LII) immune infiltrate and high and low (HF and LF) functions. Consistent with this idea, the LF expressed specific proliferative genes (MAPK and E3 ubiquitin ligase) and aggressive behaviour. On the other hand, HF exhibited enriched immune gene pathways with an inflammatory profile, including genes related to immune cell recruitment, as well as, adaptive and innate immunity. The authors suggested a profile infiltrate and functional transcriptomic integration with four different subtypes in the TME: LII-LF, LII-HF, HII-HF and HII-LF. Under these circumstances, it is relevant because the H-MSI and CIN are compatible with HII-HF and highly express the inhibitor molecules (PD1, PD-L1 and CTL4) implicated in immunotherapy response (Figure 2C).

**Figure 3.**
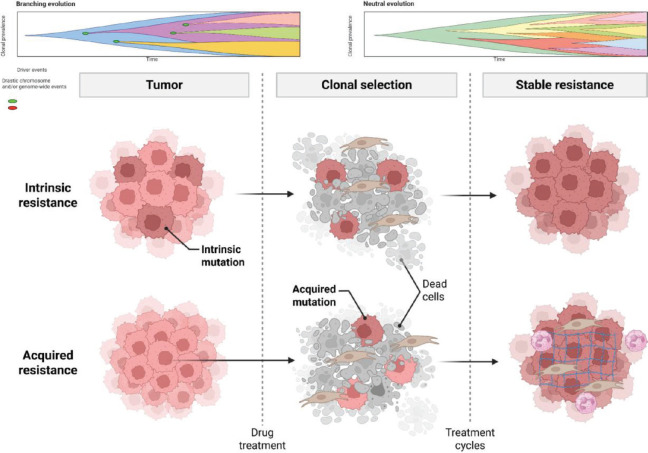
The GC TME, which has critical components such as immune cells, stromal extracellular matrix and clonal tumor plasticity, contributes to high heterogeneity and immunosuppressiveness. The cellular and molecular interactions and communication between these TME actors determine the partial response to immunotherapy. Other elements, such as metabolites and proteins, are also involved in the immunosuppressive state; however, these complex dynamics are challenging to understand. In this context, the spatial omics attempt to improve our knowledge of GC TME and characterisation of these three components play a fundamental role because they favour the resistance to chemotherapy and immunotherapy in GC. It is important because development target therapy for its TME modulation is the objective in the future research. Created with www.bioRender.com.

The high plasticity of GC is underscored by autophagy-related genes MARCKS and TXNIP, which are associated with a poor prognosis [82]. This is significant because the high heterogeneity can partly explain the plasticity of cells and therapy resistance [83]. Moreover, stem cells are provided to heterogeneous cell subpopulations, biological characteristics, pathological features and treatment sensitivity according to the context of regional differences. The GC cell’s heterogeneity and complexities result from clonal evolution and are driven by genetic instability, TME pressures, hostility with polyclonal differentiation and stochastic genetic and epigenetic changes [83]. This phenomenon is noteworthy because it constitutes a hallmark of cancer [84]. Similarly, it has been shown that GC clinical behaviour is heterogeneous, and partial response to treatment results from high histological, transcriptomic and epigenomic expression and variation, with multiple distinct subtypes in a unique tumour that has undergone dynamic and transformational changes in the TME (Figure 3) [11, 35].

The phenotype of gastric epithelial cells is reshaped through transdifferentiation, dedifferentiation and blocked differentiation, exhibiting distinct differentiation phenotypic characteristics. The plasticity of GC is derived from multiple factors, including activated transcription factors, abnormal epigenetic regulation, chronic *Helicobacter pylori* infection, an acidic environment and the activation of canonical signaling pathways, such as WNT/B-catenin, notch signaling pathway (NOTCH) and Sonic Hedgehog [85].

Intratumoural heterogeneity has been linked to the emergence of tumour subclones and is a source of therapeutic resistance [72, 86]. In this context, intratumoural heterogeneity studies have utilised DNA-based genomic sequencing to identify clonal and subclonal genomic alterations, including mutations and sCNA, thereby enabling the phylogenetic reconstruction of tumour evolutionary trajectories [72, 87–92]. Of note, the timeline and architecture of intratumoural evolution have been uncovered in tumour cellular states associated with subclone evolution and interaction with TME [93].

The understanding of heterogeneity and its plasticity, a key role in GC TME, has been facilitated by spatial-omics tools, and their implications for immunotherapy and immune response are peremptory for developing and focusing novel targeted therapies for GC [11, 12, 17, 83] (Figure 3).

On the other hand, the Tregs are a master cell in the GC TME, and their fundamental role is related to immune evasion. Recent studies with spatial transcriptomics showed a critical distribution in TME that can improve the understanding of immune response and precision of immunotherapy [94].

Finally, we discuss the cancer-associated fibroblast (CAF) subpopulations and their role in regulating dynamic communication between ECM, TME, immune cells, stromal cells and other cell types [36]. The CAF plus SPP1 (multifunctional cytokine) plays a crucial role in intratumoural heterogeneity, and SPP1/CD44+ is involved in the progression of GC [95]. Additionally, the ﻿SPP1/CD44+ axis is critical to the interaction between TAM and GC cells within the TME, enabling the activation of cancer-associated signaling pathways [95].

## Integrated-spatial omics enabling novel targeted therapy

The spatial-omics applications can enable the TME characterisation and prediction, allowing for an enhanced understanding of TME ImmunoBiology in GC. This is essential because refining this knowledge is essential in the search for novel target therapies. In this scenario, a study of the GC murine model found that the combination of anti-IL17 and anti-PDL1 was associated with tumour regression [96]. Moreover, another study with spatial transcriptomic analysis (NanoString nCounter System and Illumina TST170 gene panel) of tumour cell specimens attempted to investigate target drug resistance genes of GC. This study showed that HER2 and FGFR2 protein expression are possible targets [97].

Similarly, crizotinib is a targeted therapy that blocks MAPK signahypoxicy and hypoxic TME, tumour angiogenesis and liver metastasis; given this, the role of play inhibits the metastatic progression of K-RA cells (-mutated) [79].

Additionally, it has been demonstrated that IL-18 signaling pathways were enriched in a functional assessment, which may contribute to the differential functional states of the GC TME [53] and its consideration as an immune response biomarker.

The comprehensive characterisation of TIME is plausible due to the immune transcriptomic profile, which can provide a prediction model for immunotherapy response, identify checkpoint inhibitor (CPI)-sensitive tumours and delineate the accuracy of beneficial patients with ICI that can complement CPI and combined positive score (CPS). Consistent with this idea, 67% of immunotherapy patient responders correspond to IIH-HF, and its classification is an exciting approach for response to immunotherapy (*p* = 0.003) and predicts response to CPS <5 [53]. These findings are valued because they enable the precise and accurate detection of patients who could benefit from immunotherapy and are complementary to CPS.

On the other hand, the study conducted by Akiyama *et al* [98], which used 10× Genomic Visium for spatial transcriptomics, successfully elucidated that PDGFRα/β dual blockade ameliorated the GC TME through ECM shaping, enhancing the anti-PDL1 antibody and immune response in the Fibrotic TME. This study is significant because TGF-β signaling in epithelial tumour cells can lead to a MES GC phenotype. We now understand that the intrinsic mechanism of MES reprogramming favours the drug resistance of GC tumour cells [85, 99, 100].

The PDGF-PDGFR targeted therapy against PDGFRβ signaling emerges as an effective target for ameliorating the Fibrotic cancer tissue matrix environment influenced by TGFβ signaling. Considering this circumstance, it’s imperative to infiltrate *T* cells so that TME surrounds the tumour cells and improves the immunotherapy response [101]. Besides, PDGF increased the expression of CXCLs involved in recruiting polymorphonuclear myeloid-derived suppressor cells [102–104]. The PDGF/PDGFR pathway is associated with cancer proliferation, metastasis, invasion and angiogenesis through modulating multiple downstream pathways, which include PI3K/AKT/PKB (Protein Kinase B)/mTOR, AKT/protein kinase B pathway, MAPK/extracellular signal-regulated kinase pathway, JAK/STAT pathway and NOTCH pathway [105].

## Future perspectives and new frontiers

Despite the significant advances made by omics sciences in understanding the TME of the GC, it is still too early to say what fundamental role it will play. However, we hope that it will play a transcendental role, as it reveals detailed analysis and information that were previously impossible to obtain. This overwhelming new knowledge will give us insight into how to continue searching for new therapies to improve survival. In this regard, this integrative effort holds excellent promise for defining disease subtypes, intratumoural and intertumoural heterogeneity, tumour resistance, predicting prognosis and enabling targeted therapies to be delivered based on the spatial distribution of specific cell subtypes. It also allows the detection of ligands and receptors involved in their mechanism of action.

**Figure 4.**
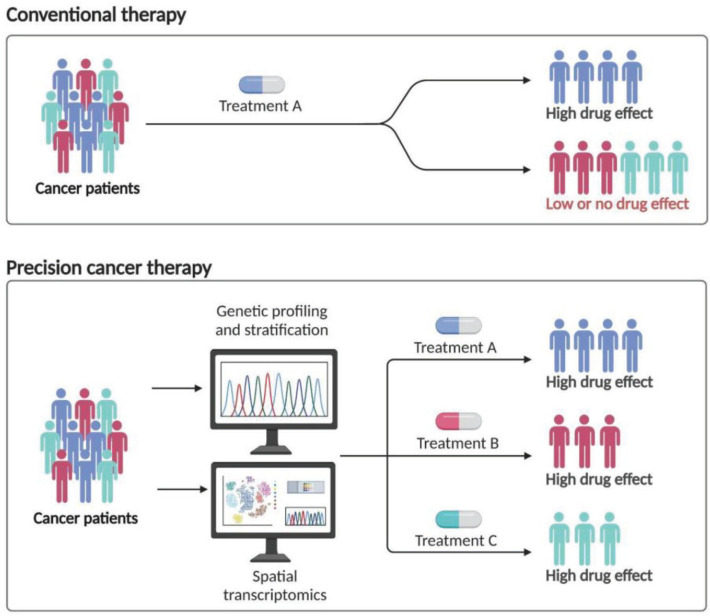
Personalised management of GC. Created with www.bioRender.com.

Immune cells, stromal cells and clonal cells are evolving as keys to understanding immunosuppressive and heterogeneous TME in the immune response to immunotherapy; furthermore, spatial omics is fundamental in the comprehensive understanding of these pillars in the search for effective therapies in precision oncology (Figure 4).

AI is characterised by deep learning, neuronal networks and the efficient processing of millions of complex data points, allowing for the classification of molecular subgroups, cellular states, intratumoural and intertumoural heterogeneity, tumour progression pathways and and cell fate [14, 15, 24, 33, 35, 53]. Under these circumstances, the headway will be straightforward and rapid, which promises advances in precision oncology and targeted therapy of GC.

The studies conducted in recent years on spatial omics technologies have made invaluable contributions to the knowledge of GC. Current advances have been reflected in the proposed new therapeutic targets. Despite this, harmful outcomes are evident in the prognosis and treatment [106]. In this effect, the search for Immunocore is indispensable for the precision assessment of current immunotherapy responses, enabling the identification of patients who are candidates for effective therapy. Spatial omics aims to characterise TIME, chemokines and cell phenotypes to explore cell interplay, signal transduction pathways and immune cell and protein trafficking. According to the above, having clearance for this landscape is vital for success in tumour-infiltrating cells (TILs) and functional TME phenotype for immune response to immunotherapy. This therapeutic approach has likely been noteable and paradigmatic progress in recent years and near future precision oncology and personalised management (Figure 4). However, significant challenges would still need to be overcome to achieve successful treatment and improve patient survival.

The main limitations of spatial omics technologies include sample preparation, the need for sophisticated and logistic, and the complex translation to clinical practice. Moreover, these platforms are expensive. In this sense, only some are commercial, such as 10× Genomics Visium, GeoMX (NanoString) and RNAScope (high-resolution targeted methods).

The integration of spatial transcriptomics with other omics disciplines, such as proteomics, epigenomics and metabolomics, will enable a more comprehensive understanding of gastric tumour biology. However, analysing the complexity of these relationships within a spatial context remains a significant challenge. In these conditions, complementary analysis using machine learning tools – particularly deep learning – can aid in the interpretation of these intricate interactions.

## Conflicts of interest

None declared.

## Funding

None.

## Patient and public involvement

Patients and/or the public were not involved in the design, conduct, reporting or dissemination plans of this research.

## Patient consent for publication

Not applicable.

## Ethical approval

Not applicable.

## Provenance and peer review

Not commissioned; externally peer reviewed.

## Author contributions

All authors conceived and drafted the manuscript.

## Figures and Tables

**Table 1.** Hallmarks of recent studies using spatial omics in the characterisation of GC TME.

Main findings	Reference
Identify TME heterogeneity in GC, highlighting niche-specific gene signatures	[26]
Combines single-cell RNA-seq and spatial transcriptomics to map cellular interactions in GC	[27]
Tumoral cells were categorized into nine distinct categories and exhibited heightened intercellular communication, particularly with matrix fibroblasts	[28]
Spatial transcriptomics unveils the correlation of macrophage infiltration with primary resistance to the Regorafenib-Avelumab combination	[29]
Cells at the interface between tumor and non-tumor tissue exhibit a transcription profile compatible with immunometabolic alterations	[30]
The co-localisation and crosstalk between GC cells and B cells significantly affect the efficacy of immunotherapy	[31]
GC spatial transcriptomes revealed functional cellular crosstalk involving multiple cell types, including the interaction between CCL2+ fibroblasts and STAT3-activated macrophages.	[32]
Gastric tumors metastatic to the brain develop a proangiogenic gene expression profile with overexpression of DMBT-1 at tumor margins.	[33]
Gastric tumors exhibit distinct ecosystems in tumor transition, signaled by increased GREM-1 expression.	[34]
Poorly cohesive carcinomas exhibit differential profiles compared with signet ring carcinoma, particularly in relation to cell cycle genes, the respiratory chain, and MMP-7	[35]
Highly infiltrating CCND1+ fibroblasts are a risk factor for GC patients and can influence the immune and chemotherapeutic efficacy of GC patients	[36]
GC patients with different parthanatos signals exhibited distinct immune microenvironment and metabolic reprogramming.	[37]
Young patients with GC exhibit overexpression of the IL-17, AGE-RAGE, and relaxin pathways	[38]
Oxyntic tumors exhibit overexpression of cyclin D1 and SPINK1	[39]
**Source:** Created by authors	

## References

[1] Bizuayehu HM, Dadi AF, Ahmed KY (2022). Burden of 30 cancers among men: global statistics in 2022 and projections for 2050 using population-based estimates. Cancer.

[2] Cancer Genome Atlas Research Network (2014). Comprehensive molecular characterization of gastric adenocarcinoma. Nature.

[3] WHO Classification Tumours Editorial Board (2019). WHO Classification of Tumours: Digestive System Tumours.

[4] Kushima R (2022). The updated WHO classification of digestive system tumours-gastric adenocarcinoma and dysplasia. Pathologe.

[5] Nagtegaal ID, Odze RD, Klimstra D (2020). The 2019 WHO classification of tumours of the digestive system. Histopathology.

[6] Smyth EC, Nilsson M, Grabsch HI (2020). Gastric cancer. Lancet.

[7] Joshi SS, Badgwell BD (2021). Current treatment and recent progress in gastric cancer. CA Cancer J Clin.

[8] Evans JA, Chandrasekhara V, ASGE Standards of Practice Committee (2015). The role of endoscopy in the management of premalignant and malignant conditions of the stomach. Gastrointest Endosc.

[9] Ajani JA, D’Amico TA, Bentrem DJ (2022). Gastric cancer, version 2.2022, NCCN clinical practice guidelines in oncology. J Natl Compr Canc Netw.

[10] Lordick F, Carneiro F, Cascinu S (2022). Gastric cancer: ESMO clinical practice guideline for diagnosis, treatment and follow-up. Ann Oncol.

[11] Zhu E, Xie Q, Huang X (2024). Application of spatial omics in gastric cancer [Internet]. Pathol - Res Pract.

[12] Ren L, Huang D, Liu H (2024). Applications of single cell omics and spatial transcriptomics technologies in gastric cancer (Review). Oncol Lett.

[13] Dzobo K (2020). Taking a full snapshot of cancer biology: deciphering the tumor microenvironment for effective cancer therapy in the oncology clinic. OMICS.

[14] Binnewies M, Roberts EW, Kersten K (2018). Understanding the tumor immune microenvironment (TIME) for effective therapy. Nat Med.

[15] Sadeghi Rad H, Monkman J, Warkiani ME (2021). Understanding the tumor microenvironment for effective immunotherapy. Med Res Rev.

[16] Waldman AD, Fritz JM, Lenardo MJ (2020). A guide to cancer immunotherapy: from T cell basic science to clinical practice. Nat Rev Immunol.

[17] Yasuda T, Wang YA (2024). Gastric cancer immunosuppressive microenvironment heterogeneity: implications for therapy development. Trends Cancer.

[18] Zavros Y, Merchant JL (2022). The immune microenvironment in gastric adenocarcinoma. Nat Rev Gastroenterol Hepatol.

[19] Derks S, De Klerk LK, Xu X (2020). Characterizing diversity in the tumor-immune microenvironment of distinct subclasses of gastroesophageal adenocarcinomas. Ann Oncol.

[20] Kim TS, Da Silva E, Coit DG (2019). Intratumoral immune response to gastric cancer varies by molecular and histologic subtype. Am J Surg Pathol.

[21] Lei Z, Tan IB, Das K (2013). Identification of molecular subtypes of gastric cancer with different responses to PI3-kinase inhibitors and 5-fluorouracil. Gastroenterology.

[22] Cristescu R, Lee J, Nebozhyn M (2015). Molecular analysis of gastric cancer identifies subtypes associated with distinct clinical outcomes. Nat Med.

[23] Ma Y, Jiang Z, Pan L (2024). Current development of molecular classifications of gastric cancer based on omics (Review). Int J Oncol.

[24] Bressan D, Battistoni G, Hannon GJ (2023). The dawn of spatial omics. Science.

[25] Longo SK, Guo MG, Ji AL (2021). Integrating single-cell and spatial transcriptomics to elucidate intercellular tissue dynamics. Nat Rev Genet.

[26] Eng CHL, Lawson M, Zhu Q (2019). Transcriptome-scale super-resolved imaging in tissues by RNA seqFISH. Nature.

[27] Chen J, Suo S, Tam PP (2017). Spatial transcriptomic analysis of cryosectioned tissue samples with Geo-seq. Nat Protoc.

[28] Vickovic S, Eraslan G, Salmén F (2019). High-definition spatial transcriptomics for in situ tissue profiling. Nat Methods.

[29] Rodriques SG, Stickels RR, Goeva A (2019). Slide-seq: a scalable technology for measuring genome-wide expression at high spatial resolution. Science.

[30] Lee JH, Daugharthy ER, Scheiman J (2015). Fluorescent in situ sequencing (FISSEQ) of RNA for gene expression profiling in intact cells and tissues. Nat Protoc.

[31] Lovatt D, Ruble BK, Lee J (2014). Transcriptome in vivo analysis (TIVA) of spatially defined single cells in live tissue. Nat Methods.

[32] Hu KH, Eichorst JP, Mcginnis CS (2020). ZipSeq: barcoding for real-time mapping of single cell transcriptomes. Nat Methods.

[33] Chen J, Xu H, Tao W (2023). Transformer for one stop interpretable cell type annotation. Nat Commun.

[34] Asp M, Bergenstråhle J, Lundeberg J (2020). Spatially resolved transcriptomes-next generation tools for tissue exploration. Bioessays.

[35] Kumar V, Ramnarayanan K, Sundar R (2022). Single-cell atlas of lineage states, tumor microenvironment, and subtype-specific expression programs in gastric cancer. Cancer Discovery.

[36] Li X, Sun Z, Peng G (2022). Single-cell RNA sequencing reveals a pro-invasive cancer-associated fibroblast subgroup associated with poor clinical outcomes in patients with gastric cancer. Theranostics.

[37] Jeongg HY, Ham IH, Lee SH (2021). Spatially distinct reprogramming of the tumor microenvironment based on tumor invasion in diffuse-type gastric cancers. Clin Cancer Res.

[38] Sundar R, Liu DH, Hutchins GG (2021). Spatial profiling of gastric cancer patient-matched primary and locoregional metastases reveals principles of tumour dissemination. Gut.

[39] Schäfer F, Tomar A, Sato S (2024). Enhanced in situ spatial proteomics by effective combination of MALDI imaging and LC-MS/MS. Mol Cell Proteomics.

[40] Mund A, Coscia F, Kriston A (2022). Deep visual proteomics defines single-cell identity and heterogeneity. Nat Biotechnol.

[41] Seferbekova Z, Lomakin A, Yates LR (2023). Spatial biology of cancer evolution. Nat Rev Genet.

[42] De Souza N, Zhao S, Bodenmiller B (2024). Multiplex protein imaging in tumour biology. Nat Rev Cancer.

[43] Angelo M, Bendall SC, Finck R (2014). Multiplexed ion beam imaging of human breast tumors. Nat Med.

[44] Keren L, Bosse M, Thompson S (2019). MIBI-TOF: a multiplexed imaging platform relates cellular phenotypes and tissue structure. Sci Adv.

[45] Black S, Phillips D, Hickey JW (2021). CODEX multiplexed tissue imaging with DNA-conjugated antibodies. Nat Protoc.

[46] Aichler M, Walch A (2015). MALDI imaging mass spectrometry: current frontiers and perspectives in pathology research and practice. Lab Invest.

[47] Erich K, Reinle K, Müller T (2019). Spatial distribution of endogenous tissue protease activity in gastric carcinoma mapped by MALDI mass spectrometry imaging. Mol Cell Proteomics.

[48] Pigaa I, Pagni F, Magni F (2023). Cytological cytospin preparation for the spatial proteomics analysis of thyroid nodules using MALDI-MSI. Methods Mol Biol.

[49] Dentt A, Diamandis P (2022). Integrating computational pathology and proteomics to address tumor heterogeneity. J Pathol.

[50] Marusyk A, Almendro V, Polyak K (2012). Intra-tumour heterogeneity: a looking glass for cancer?. Nat Rev Cancer.

[51] Saka SK, Wang Y, Kishi JY (2019). Immuno-SABER enables highly multiplexed and amplified protein imaging in tissues. Nat Biotechnol.

[52] Kinkhabwala A, Herbel C, Pankratz J (2022). MACSima imaging cyclic staining (MICS) technology reveals combinatorial target pairs for CAR T cell treatment of solid tumors. Sci Rep.

[53] Cabeza-Segura M, Gambardella V, Gimeno-Valiente F (2022). Integrative immune transcriptomic classification improves patient selection for precision immunotherapy in advanced gastro-oesophageal adenocarcinoma. Br J Cancer.

[54] Karczewskii KJ, Snyder MP (2018). Integrative omics for health and disease. Nat Rev Genet.

[55] Karacosta LG (2021). From imaging a single cell to implementing precision medicine: an exciting new era. Emerg Top Life Sci.

[56] He MJ, Pu W, Wang X (2022). Comparing DESI-MSI and MALDI-MSI mediated spatial metabolomics and their applications in cancer studies. Front Oncol.

[57] Buck A, Ly A, Balluff B (2015). High-resolution MALDI-FT-ICR MS imaging for the analysis of metabolites from formalin-fixed, paraffin-embedded clinical tissue samples. J Pathol.

[58] Van De Plas R, Yang J, Spraggins J (2015). Image fusion of mass spectrometry and microscopy: a multimodality paradigm for molecular tissue mapping. Nat Methods.

[59] Wang L, Xing X, Zeng X (2022). Spatially resolved isotope tracing reveals tissue metabolic activity. Nat Methods.

[60] Zhang H, Lu KH, Ebbini M (2024). Mass spectrometry imaging for spatially resolved multi-omics molecular mapping. Npj Imag.

[61] Nunes JB, Ijsselsteijn ME, Abdelaal T (2024). Integration of mass cytometry and mass spectrometry imaging for spatially resolved single-cell metabolic profiling. Nat Methods.

[62] Ly A, Longuespée R, Casadonte R (2019). Site-to-site reproducibility and spatial resolution in MALDI-MSI of peptides from formalin-fixed paraffin-embedded samples. Proteomics Clin Appl.

[63] Xie T, Huang A, Yan H (2024). Artificial intelligence: illuminating the depths of the tumor microenvironment. J Translational Med.

[64] Chon HJ (2024). Artificial intelligence (AI)-powered tumor microenvironment (TME) analysis to identify potential biomarkers for ICIs with or without bevacizumab in hepatocellular carcinoma (HCC) [Internet]. JCO.

[65] Malherbee K (2021). Tumor microenvironment and the role of artificial intelligence in breast cancer detection and prognosis. Am J Pathol.

[66] Bagaev A, Kotlov N, Nomie K (2021). Conserved pan-cancer microenvironment subtypes predict response to immunotherapy. Cancer Cell.

[67] Davis-Marcisak EF, Deshpande A, Stein-O’Brien GL (2021). From bench to bedside: single-cell analysis for cancer immunotherapy. Cancer Cell.

[68] Zheng L, Qin S, Si W (2021). Pan-cancer single-cell landscape of tumor-infiltrating T cells. Science.

[69] Wu Y, Yang S, Ma J (2022). Spatiotemporal immune landscape of colorectal cancer liver metastasis at single-cell level. Cancer Discov.

[70] Peng Z, Ren Z, Tong Z (2023). Interactions between MFAP5 + fibroblasts and tumor-infiltrating myeloid cells shape the malignant microenvironment of colorectal cancer. J Translational Med.

[71] Marx V (2021). Method of the year: spatially resolved transcriptomics. Nat Methods.

[72] Ma H, Srivastava S, Ho SWT (2025). Spatially resolved tumor ecosystems and cell states in gastric adenocarcinoma progression and evolution. Cancer Discov.

[73] Li Y, Hu X, Lin R (2022). Single-cell landscape reveals active cell subtypes and their interaction in the tumor microenvironment of gastric cancer. Theranostics.

[74] Depeaux K, Delgoffe GM (2021). Metabolic barriers to cancer immunotherapy. Nat Rev Immunol.

[75] Fu K, Hui B, Wang Q (2020). Single-cell RNA sequencing of immune cells in gastric cancer patients. Aging (Albany NY).

[76] Sundar R, Huang KK, Kumar V (2022). Epigenetic promoter alterations in GI tumour immune-editing and resistance to immune checkpoint inhibition. Gut.

[77] Li S, Li K, Tian F (2022). A High interferon gamma signature of CD8+ T cells predicts response to neoadjuvant immunotherapy plus chemotherapy in gastric cancer. Front Immunol.

[78] Sathe A, Grimes SM, Lau BT (2020). Single-cell genomic characterization reveals the cellular reprogramming of the gastric tumor microenvironment. Clin Cancer Res.

[79] Yamasaki J, Hirata Y, Otsuki Y (2022). MEK inhibition suppresses metastatic progression of KRAS-mutated gastric cancer. Cancer Sci.

[80] Zheng GX, Terry JM, Belgrader P (2017). Massively parallel digital transcriptional profiling of single cells. Nat Commun.

[81] Shah M, Sarkar S, Melikhova D (2024). Gastric cancer tumor immune microenvironment landscape: clinical implications for patient outcome and response to PD-1 immune checkpoint blockade. Res Sq.

[82] Huang XZ, Pang MJ, Li JY (2023). Single-cell sequencing of ascites fluid illustrates heterogeneity and therapy-induced evolution during gastric cancer peritoneal metastasis. Nat Commun.

[83] Niu X, Liu W, Zhang Y (2024). Cancer plasticity in therapy resistance: mechanisms and novel strategies. Drug Resistant Updates.

[84] Hanahan D (2022). Hallmarks of cancer: new dimensions. Cancer Discov.

[85] Yan Z, Liu Y, Yuan Y (2024). The plasticity of epithelial cells and its potential in the induced differentiation of gastric cancer. Cell Death Discovery.

[86] Cajal RYS, Sesé M, Capdevila C (2020). Clinical implications of intratumor heterogeneity: challenges and opportunities. J Mol Med (Berl).

[87] Deshwar AG, Vembu S, Yung CK (2015). PhyloWGS: reconstructing subclonal composition and evolution from whole-genome sequencing of tumors. Genome Biol.

[88] Coorens THH, Spencer Chapman M, Williams N (2024). Reconstructing phylogenetic trees from genome-wide somatic mutations in clonal samples. Nat Protoc.

[89] Lee YS, Cho YS, Lee GK (2014). Genomic profile analysis of diffuse-type gastric cancers. Genome Biol.

[90] Zhang J, Qiu W, Liu H (2018). Genomic alterations in gastric cancers discovered via whole-exome sequencing. BMC Cancer.

[91] Zhu Z, Fu H, Wang S (2020). Whole-exome sequencing identifies prognostic mutational signatures in gastric cancer. Ann Transl Med.

[92] Fu YC, Liang SB, Luo M (2025). Intratumoral heterogeneity and drug resistance in cancer. Cancer Cell Int.

[93] Vitale I, Sistigu A, Manic G (2019). Mutational and antigenic landscape in tumor progression and cancer immunotherapy. Trends Cell Biol.

[94] Wang B, Zhang Z, Liu W (2023). Targeting regulatory T cells in gastric cancer: pathogenesis, immunotherapy, and prognosis. Biomed Pharmacother.

[95] Xie W, Cheng J, Hong Z (2023). Multi-transcriptomic analysis reveals the heterogeneity and tumor-promoting role of SPP1/CD44-mediated intratumoral crosstalk in gastric cancer. Cancers (Basel).

[96] Nagaoka K, Shirai M, Taniguchi K (2020). Deep immunophenotyping at the single-cell level identifies a combination of anti-IL-17 and checkpoint blockade as an effective treatment in a preclinical model of data-guided personalized immunotherapy. J Immunother Cancer.

[97] Kleo K, Jovanovic VM, Arndold A (2023). Response prediction in patients with gastric and esophagogastric adenocarcinoma under neoadjuvant chemotherapy using targeted gene expression analysis and next-generation sequencing in pre-therapeutic biopsies. J Cancer Res Clin Oncol.

[98] Akiyama T, Yasuda T, Uchihara T (2023). Stromal reprogramming through dual PDGFRα/β blockade boosts the efficacy of anti-PD-1 immunotherapy in fibrotic tumors. Cancer Res.

[99] Saitoh M (2015). Epithelial–mesenchymal transition is regulated at post-transcriptional levels by transforming growth factor-β signaling during tumor progression. Cancer Sci.

[100] Jollyy MK, Somarelli JA, Sheth M (2019). Hybrid epithelial/mesenchymal phenotypes promote metastasis and therapy resistance across carcinomas. Pharmacol Ther.

[101] Pandeyy P, Khan F, Upadhyay TK (2023). New insights about the PDGF/PDGFR signaling pathway as a promising target to develop cancer therapeutic strategies. Biomed Pharmacother.

[102] Raskov H, Orhan A, Gaggar S (2022). Neutrophils and polymorphonuclear myeloid-derived suppressor cells: an emerging battleground in cancer therapy. Oncogenesis.

[103] Calderon JJ, Prieto K, Lasso P (2023). Modulation of myeloid-derived suppressor cells in the tumor microenvironment by natural products. Arch Immunol Ther Exp.

[104] Portillo-Miño JD, Calderón JJ, Ruiz-García E (2025). Myeloid-derived suppressor cells modulation in the context of tumor microenvironment for gastric cancer. Clin Transl Oncol.

[105] Zou X, Tang XY, Qu ZY (2022). Targeting The PDGF/PDGFR signaling pathway for cancer therapy: a review. Int J Biol Macromol.

[106] Fridman WH, Zitvogel L, Sautès–Fridman C (2017). The immune contexture in cancer prognosis and treatment. Nat Rev Clin Oncol.

